# The Efficacy of Surfactant Replacement Therapy in the Growth-Restricted Preterm Infant: What is the Evidence?

**DOI:** 10.3389/fped.2014.00118

**Published:** 2014-10-29

**Authors:** Atul Malhotra, Arun Sasi, Suzanne L. Miller, Graham Jenkin, Graeme R. Polglase

**Affiliations:** ^1^Monash Newborn, Monash Children’s Hospital, Melbourne, VIC, Australia; ^2^The Ritchie Centre, Monash Institute of Medical Research, Melbourne, VIC, Australia; ^3^Department of Paediatrics, Monash University, Melbourne, VIC, Australia; ^4^Department of Obstetrics and Gynaecology, Monash University, Melbourne, VIC, Australia

**Keywords:** premature, IUGR, fetal growth restriction, FGR, lungs, RDS, BPD

## Abstract

**Background:** Surfactant replacement therapy (SRT) is an integral part of management of preterm surfactant deficiency respiratory distress syndrome (RDS). Its role in the management of RDS has been extensively studied. However, its efficacy in the management of lung disease in preterm infants born with intrauterine growth restriction (IUGR) has not been systematically studied.

**Objective:** To evaluate the efficacy of exogenous SRT in the management of preterm IUGR lung disease.

**Methods:** A systematic search of all available randomized clinical trials (RCT) of SRT in preterm IUGR infants was done according to the standard Cochrane collaboration search strategy. Neonatal respiratory outcomes were compared between the preterm IUGR and appropriately grown for gestational age (AGA) preterm infant populations in eligible studies.

**Results:** No study was identified which evaluated the efficacy or responsiveness of exogenous SRT in preterm IUGR infants as compared to preterm AGA-infants. The only study identified through the search strategy used small for gestational age (SGA; defined as less than tenth centile for birth weight) as a proxy for IUGR. The RCT evaluated the efficacy or responsiveness of SRT in preterm SGA group as compared to AGA-infants. The rate of intubation, severity of RDS, rate of surfactant administration, pulmonary air leaks, and days on the ventilator did not differ between both groups. However, the requirement for prolonged nasal continuous positive airway pressure (*p* < 0.001), supplemental oxygen therapy (*p* < 0.01), and the incidence of bronchopulmonary dysplasia at 28 days and 36 weeks (both *p* < 0.01) was greater in SGA-infants.

**Discussion:** There is currently insufficient data available to evaluate the efficacy of SRT in preterm IUGR lung disease. A variety of research strategies will be needed to enhance our understanding of the role and rationale for use of SRT in preterm IUGR lung disease.

## Introduction

Introduction of surfactant replacement therapy (SRT) in the 1990s marked the beginning of a new era in the management of prematurity-related surfactant deficiency and its clinical sequelae, respiratory distress syndrome (RDS) (1). Today, SRT, along with antenatal glucocorticoid therapy, are recognized as the principal interventions that decrease the overall mortality in preterm infants. Further, the rationale for treatment is well supported by robust scientific evidence (2, 3). SRT reduces the inspired oxygen and ventilation requirements of the neonate, as well as the incidence of severe RDS, pneumothorax, pulmonary interstitial emphysema, and death (4, 5). Refinements in treatment strategies, including timing of surfactant therapy, choice of preparation, number of doses, and techniques for administration have been established (6, 7).

Intrauterine growth restriction and prematurity are two important pregnancy complications that lead to high rates of mortality and morbidity in neonates. Unfortunately, they also commonly co-exist, and thereby potentiate the risk of adverse outcomes (8). Intrauterine growth restriction (IUGR) complicates 10–25% of preterm births and its incidence increases with decreasing gestational age at delivery (9–11). Abnormal intrauterine cardiovascular, neuroendocrine, metabolic, and oxidative stress states in growth-restricted infants make them susceptible to both short- and long-term complications (12). Compared to appropriately grown age-matched counterparts, IUGR babies have higher mortality rates, are at significant risk for reduced postnatal growth and development, and have increased incidence of morbidities such as RDS, bronchopulmonary dysplasia (BPD), retinopathy of prematurity, and necrotizing enterocolitis (13–15).

Despite differences in postnatal sequelae for IUGR vs. normally grown babies, current respiratory management of lung disease in preterm IUGR infants is not different to that of preterm AGA-infants, which includes supportive respiratory care, early initiation of continuous positive airway pressure (CPAP), and early rescue surfactant therapy if RDS develops or worsens.

The purpose of this review is to systematically explore the evidence available for the efficacy of exogenous surfactant therapy in the treatment of preterm IUGR lung disease. There is evidence to suggest from animal studies that preterm IUGR lung disease may be different to the “classical” or “modern” standard preterm lung disease that is observed in preterm AGA-infants. To this effect, we intend to refresh our understanding of the science behind preterm IUGR lung disease, with emphasis on the effect of IUGR on *endogenous* surfactant production, metabolism, and how *exogenous* SRT may influence this. We aim to examine the influence of IUGR on the pulmonary outcomes in the *preterm neonate*, and generate research frontiers in this relatively poorly described area of neonatal medicine.

## Methods

For purposes of this review, studies of preterm infants diagnosed with IUGR were included in the study group. IUGR by definition needs to have a series of prenatal weight evaluations showing a drop in weight centiles. Generally, the observed weight is less than tenth centile for that gestation with ultrasound Doppler evidence of abnormal placental flow. The control group comprised of preterm AGA-infants.

### Criteria for considering studies for the meta-analysis

#### Eligibility

Randomized or quasi-randomized controlled clinical trials comparing efficacy of surfactant administration in the form of respiratory outcomes of IUGR infants to outcomes in AGA-infants <37 weeks of gestation, at risk or with established RDS were considered for review.

#### Search strategy

Standard Cochrane Neonatal Review Group (CNRG) search criteria were used to identify relevant studies. We searched the Cochrane Central Register of Controlled Trials (CENTRAL, The Cochrane Library, 2014, Issue 1), MEDLINE (MeSH terms: pulmonary surfactant AND fetal growth restriction OR IUGR; limits: age, newborn: publication type: clinical trial), EMBASE, CINAHL, conference abstracts from Pediatric Academic Societies, and journal hand searching in the English language. A search was also made on “IUGR” and “respiratory outcomes” using the same limits as above. Two authors (Atul Malhotra and Arun Sasi) independently conducted the search and evaluated the articles for eligibility, methodological quality, and risk of bias.

#### Outcomes

Outcomes included mortality (prior to 28 days of age, discharge), BPD (oxygen requirement at 28 days of age, 36 weeks post menstrual age), death or BPD (prior to 28 days, 36 weeks post menstrual age), duration of ventilation, and duration supplemental oxygen use. Secondary outcomes included air leaks, pulmonary hemorrhage, culture proven sepsis, patent ductus arteriosus, intraventricular hemorrhage, periventricular leukomalacia, retinopathy of prematurity, necrotizing enterocolitis, and days of hospital stay.

#### Data collection and analysis

Data collection was done using the standard methods of the CNRG. For each trial, information was extracted regarding method of randomization, allocation concealment strategies, and completeness of reporting. The data were analyzed using RevMan5 (v5.2 2014, Copenhagen; The Nordic Cochrane Center, Cochrane Collaboration 2012).

## Results

The preliminary MEDLINE search (IUGR and surfactant) yielded a total of 48 studies as per the search criteria. A repeat search (IUGR and respiratory outcomes) yielded 59 studies. Following a detailed inspection of each of these studies, no studies were identified, which evaluated the efficacy or responsiveness of exogenous SRT in preterm IUGR infants as compared to preterm AGA-infants. The only clinical trial randomized clinical trials (RCT) (16) identified using the above search strategy evaluated the efficacy or responsiveness of SRT in the preterm small for gestational age (SGA) using SGA as a proxy for IUGR. Further examination in other search portals did not reveal additional studies and hence a meta-analysis could not be undertaken.

The included study itself was part of a randomized multicentre trial on timing of bovine surfactant therapy (17); SGA-infants were classified as infants weighing below the tenth percentile at birth and were compared to AGA-infants in terms of prenatal and neonatal characteristics and neonatal outcomes. A total of 317 infants were enrolled, 59 SGA- and 258 AGA-infants. Both groups did not differ in gestational age; however, SGA-infants had a lower birth weight (*p* < 0.001). Preterm premature rupture of fetal membranes was observed more frequently in AGA-, preeclampsia in SGA-infants. The rate of intubation, severity of RDS, rate of surfactant administration, pulmonary air leaks, and days on the ventilator did not differ between both groups. However, prolonged nasal CPAP (0 vs. 2 days, *p* < 0.001), supplemental oxygen therapy (3 vs. 6 days, *p* < 0.01), and BPD at 28 days (20.2 vs. 37.3%, *p* < 0.01) and 36 weeks (4.2 vs. 13.6%, *p* < 0.01) was diagnosed more often in SGA-infants. Furthermore neonatal mortality (0.8 vs. 10.2%, *p* < 0.0001) was significantly higher in SGA-infants.

The overall RCT from which the sub-group analysis was conducted for the included study had adequate random sequence generation, allocation concealment. Further analysis was not conducted given the lack of any other relevant studies.

## Discussion

The review was conducted to systematically evaluate the effect of exogenous surfactant on preterm IUGR lung disease. We could find no suitable preterm IUGR study for this evaluation and hence a meta-analysis and inference cannot be made based on available evidence. The only relevant study included infants who were SGA at birth, not those who were diagnosed as IUGR. This highlights an important issue with literature on this subject and a lack of standard definitions. SGA describes small infants, <10% centile for birth weight, but these infants may be otherwise well but constitutionally small. Whereas, IUGR is generally characterized by the presence of serial evaluations of falling fetal growth, commonly characterized by descriptive indices of <10% centile for weight at particular gestation and abnormal umbilical Doppler ultrasound. Clear definitions are not always provided in published work, and some studies on IUGR infants may be simply called SGA, and vice versa.

We present the current understanding of preterm IUGR lung disease first and then discuss the role of exogenous surfactant.

### Preterm lung disease in growth-restricted infants

Preterm infants with IUGR have a high risk of pulmonary morbidities including long-term alterations in respiratory function (18–20), however, the effect of IUGR on incidence and severity of RDS is still debatable. It is important to reflect on the diagnosis of RDS in this era of wide-spread antenatal steroid use and early non-invasive respiratory support. Unless there is chest X-ray evidence of RDS, the diagnosis of RDS in every preterm infant with distress, and especially in an IUGR infant, may be fraught with error and misdiagnosis. The perceived notion of accelerated lung maturation and, hence, reduced incidence and severity of RDS in IUGR infants are refuted by a number of studies (21–23). McIntire and co-workers reported an increasing incidence of RDS with decreasing birth weight centiles, at any given gestational age. Infants born at ≤25 weeks gestation had a higher incidence of RDS compared to heavier infants at all gestational ages between 28 to 37 weeks (22). Spinillo et al. similarly reported a significantly increased risk of RDS in infants born between 24 to 31 weeks gestation with IUGR (24). Several underlying mechanisms have been postulated to explain this increased incidence of RDS in IUGR infants, such as reduced or impaired surfactant release or diminished response to glucocorticoids (12).

An additional concern for IUGR infants is the observed association between IUGR and BPD (13, 25, 26). BPD may occur as a consequence of high levels of oxygen or prolonged use of invasive ventilation, wherein the still relatively immature lungs become damaged and normal lung development is interrupted. Compared to AGA-infants, growth-restricted newborns have a three to fourfold increase in oxygen dependency at 36 weeks postmenstrual age (26). It is however poorly understood why IUGR infants have high rates of BPD compared to their AGA counterparts. It may be hypothesized that the underlying susceptibility for BPD in IUGR babies reflects a continuum of adverse events affecting lung development, with its origins in fetal life (27).

*In utero*, IUGR fetuses must adapt to chronic hypoxia to survive. One key adaptation is the redistribution of cardiac output to preferentially provide blood flow and oxygen supply to the brain and heart, commonly referred to as “brain-sparing.” While this response may assist survival *in utero*, it necessitates reduced oxygen supply to other organs during development, including the lungs (28, 29). In experimental sheep studies of IUGR fetuses and offspring, growth restriction induced abnormal lung structure and functional deficits in gas exchange, and reduced lung compliance, and these functional lung deficits persisted to adolescence (30, 31). It is, however, not known whether basic lung developmental alterations underlie the vulnerability of the IUGR infant to BPD, particularly with the knowledge that IUGR is also associated with a variety of metabolic, cardiovascular, and endocrine dysfunctions that could in turn lead to oxygen free radical and inflammatory imbalance, reperfusion injury, and therefore more severe or greater susceptibility to early neonatal lung disease (31–33).

### Effect of IUGR on endogenous surfactant

Understanding the basic physiology of the IUGR lung, and differences with control lungs in relation to endogenous surfactant is required to appreciate potential differences in the requirement and/or response to exogenous surfactants. The scientific data for the effect of IUGR on endogenous surfactant production and function is sparse and conflicting. Whereas some experimental studies in animal models reported significant alteration in maturation of Type II pneumocytes resulting in reduced surfactant content and activity others have reported a significant increase in surfactant protein synthesis (34, 35). Furthermore, chronic hypoxia and acidosis associated with IUGR may attenuate surfactant synthesis (27). In a sheep model of IUGR induced by placental embolization from 120 to 140 days gestation, Cock et al. did not demonstrate any alteration in surfactant protein mRNA expression of SP-A, B, or C in lung tissue at term (36). In contrast, Gagnon et al. reported increased lung tissue SP-A and SP-B mRNA expression, and that the increase was closely associated with plasma cortisol levels. Chronic fetal growth restriction from conception in carunclectomised sheep showed decreased lung tissue mRNA expression of SP-A, B, and C at 133 and 141 days gestation, with an inverse relationship between plasma cortisol concentration and SP-A and B expression (37). In contrast, IUGR induced by single umbilical artery ligation in sheep fetuses from 108 days gestation had no effect on lung tissue surfactant mRNA levels by 115 days gestation, nor did it alter alveolarisation (38). In addition to observed alterations in surfactant protein synthesis, fetal growth restriction could also affect the lipid components of surfactant. For example, growth-restricted rats are shown to have significantly reduced phosphatidylcholine content, lower lung volume, and reduced alveolar air spaces (39). Importantly, none of these studies have looked at levels of surfactant protein in the airways (e.g., in bronchoalveolar lavage fluid), the composition of the surfactant produced, the number of lamellar bodies within the type 2 alveolar epithelial cells, or the ability of surfactant release from the lamellar bodies (surfactant availability). These experimental observations, when available, would assist in understanding surfactant release, turnover, and metabolism at the alveolar level in IUGR and AGA fetuses.

### Exogenous surfactant in growth-restricted preterm lung disease

We did not identify any studies that evaluated surfactant response in preterm IUGR infants. One study evaluated the responsiveness to surfactant therapy in preterm SGA-infants, as discussed above. The outcomes in this preterm SGA cohort were different as compared to the AGA group, but reflected more chronic outcomes such as oxygen use, BPD, mortality, and length of stay as compared to acute outcomes (days of ventilation, pulmonary air leaks, pulmonary hemorrhage, and IVH). Whilst studies have shown superior benefits of surfactant therapy in a sub-group of premature infants <1000 g, there are no studies that have specifically looked at the effectiveness of surfactant therapy in IUGR infants alone (3–5). As described above, whether there is a strong association between preterm lung disease and elevated risk in IUGR infants has not yet been conclusively described, and there are not yet definitive animal studies in appropriate IUGR models. But, there are now strong indications that pulmonary function and structure in preterm IUGR infants appear quite different compared to appropriately grown babies. With this in mind it should be queried whether the management strategy of treating all RDS infants with a blanket approach is best-practice. Alterations in basic morphology, lung maturation, and/or pulmonary surfactant functions induced by fetal growth restriction would be expected to influence the effectiveness of exogenous surfactant when administered to IUGR preterm infants. Considering the demonstrated impact of growth restriction on the immature lung, it is logical to hypothesize that exogenous surfactant responsiveness as well as the ideal dose; number of doses etc. might be different in IUGR compared to AGA babies. Furthermore, such treatment may be influenced by the degree of growth restriction.

Indeed, most studies related to surfactant use in preterm infants have not reported a sub-group analysis on the effectiveness of surfactant therapy in IUGR infants (3, 4). The relatively small numbers of IUGR infants with the potential to be recruited into a larger study significantly impacts the availability of data for this sub-group. However, it is interesting, and perhaps unfortunate that the animal literature is also sparse in terms of information on the effect of postnatal surfactant administration in IUGR newborns (20, 22). There is some indirect experimental data that alludes to the effects of surfactant on IUGR lungs, using analysis of tissue surfactant content but even these results are conflicting, and are dependent upon the species, the method used to induce IUGR, and the timing of the study. In a rat model of IUGR induced by bilateral uterine artery ligation, growth restriction is associated with significantly reduced surfactant apo-protein A-D levels (40). Contrary to this, a study in humans by Brianna et al. demonstrated increased circulating levels of surfactant protein D (SP-D) on postnatal day 1 and 4 (41). The authors suggest that higher SP-D concentration may reflect the relative immaturity of the lung and leaky alveolar-capillary membrane. It has also been shown that both exogenous and endogenous surfactant can be inactivated by a number of factors such as serum proteins, hemoglobin, and meconium (42, 43). *In vitro* studies show that addition of serum to the sub-phase, or mixing serum with surfactant reduces the surface activity of surfactants (44). In excised rat lungs, or in live rats, introduction of serum proteins into the trachea causes changes in mechanical behavior of the lungs consistent with surfactant inactivation (45). Inactivation of surfactant by capillary to alveolar leak of serum is implicated in the pathogenesis of acute lung injury (46). Akin to acute lung injury, structural immaturity of lungs in the IUGR fetus leading to leaky capillary beds could in turn have a direct effect on surfactant deactivation. Presently, this cannot be supported or refuted with respect to the IUGR infant from the available literature, but this may represent another causal mechanism of lung injury and disease in IUGR infants that requires investigation.

### Research implications

The efficacy of exogenous surfactant administration in preterm IUGR infants currently represents a significant knowledge gap in neonatal research and clinical practice. There is little evidence on the effectiveness of exogenous surfactant therapy in preterm IUGR infants, primarily due to the relatively low numbers of such infants born into NICUs, and the tendency in published studies to group IUGR preterm infants with their appropriately grown counterparts. Because of this, current neonatal practice of the respiratory management of preterm IUGR infants is based on the evidence that RDS may be similar or greater in these infants as compared to AGA preterm infants. Given the ethical implications of withholding surfactant therapy from preterm IUGR infants who display features of RDS, there is a real need to turn to appropriate animal models to study efficacy of SRT. In the first instance, a greater understanding of the consequences of IUGR on surfactant production, release, and turnover will help guide future directions in the administration of exogenous surfactant therapy for these infants. The next step should be a systematic evaluation of the effects of exogenous SRT on the surfactant pool and its effect on the physiological characteristics of pulmonary function. In the future, it would be appropriate for clinical trials of surfactant replacement to address different time points, frequency, type, and nature of surfactant replacement specifically in the preterm IUGR infant population.

## Conclusion

There is insufficient data to conclude on the efficacy of SRT in management of preterm IUGR lung disease. There is strong clinical and experimental data to indicate that IUGR adversely affects lung maturation, lung function, surfactant production, and its composition in preterm neonates. There is a need for basic, as well as translational research in this area to further understand the dynamics of surfactant synthesis and function in preterm infants with IUGR to enable us to optimize respiratory care in them.

## Conflict of Interest Statement

The authors declare that the research was conducted in the absence of any commercial or financial relationships that could be construed as a potential conflict of interest.
